# Repeat transcatheter aortic valve implantation using a latest generation balloon-expandable device for treatment of failing transcatheter heart valves

**DOI:** 10.1186/s13019-016-0398-y

**Published:** 2016-01-15

**Authors:** Andreas Schaefer, Hendrik Treede, Moritz Seiffert, Florian Deuschl, Niklas Schofer, Yvonne Schneeberger, Stefan Blankenberg, Hermann Reichenspurner, Ulrich Schaefer, Lenard Conradi

**Affiliations:** Department of Cardiovascular Surgery, University Heart Center Hamburg, Martinistraße 52, D-20246 Hamburg, Germany; Department of General and Interventional Cardiology, University Heart Center Hamburg, Martinistraße 52, Hamburg, D-20246 Germany

**Keywords:** Aortic valve disease, Aortic valve stenosis, Paravalvular leak, Percutaneous valve therapy, Structural heart disease intervention, TAVI, THV

## Abstract

**Background:**

Paravalvular leakage (PVL) is a known complication of transcatheter aortic valve implantation (TAVI) and is associated with poor outcome. Besides balloon-post-dilatation, valve-in-valve (ViV) procedures can be taken into consideration to control this complication. Herein we present initial experience with use of the latest generation balloon-expandable Edwards Sapien 3® (S3) transcatheter heart valve (THV) for treatment of failing THVs.

**Methods:**

Between 01/2014 and 12/2014 three patients (two male, age: 71–80 y, log EUROScore I: 11.89 – 32.63) with failing THVs were refered to our institution for further treatment. THV approach with secondary implantation of an S3 was chosen after mutual agreement of the local interdisciplinary heart team at an interval of 533–1119 days from the index procedure. The performed procedures consisted of: S3 in Sapien XT, JenaValve and CoreValve.

**Results:**

Successful transfemoral implantation with significant reduction of PVL was achieved in all cases. No intraprocedural complications occured regarding placement of the S3 with a postprocedural effective orifice area (EOA) of 1.5–2.5 cm^2^ and pressure gradients of max/mean 14/6–36/16 mmHg. 30-day mortality was 0 %. At the latest follow-up of 90–530 days, all patients are alive and well with satisfactory THV function. Regarding VARC-2 criteria one major bleeding and one TIA was reported.

**Conclusions:**

In the instance of moderate or severe aortic regurgitation after TAVI, S3 ViV deployment is an excellent option to reduce residual regurgitation to none or mild. For further assertions concerning functional outcomes long-term results have to be awaited.

## Background

Transcatheter aortic valve implantation (TAVI) has found widespread acceptance as alternative to surgical aortic valve replacement (SAVR) over the last decade. Through introduction of transcatheter heart valves (THV) into clinical daily routine, mortality of inoperable or high-risk patients was significantly reduced compared to medical therapy [1, 2]. In 2012, the PARTNER trial presented data which showed comparable results of the first generation Edwards Sapien THV (Edwards Lifesciences Co., Irvine, CA, USA) and SAVR for severe aortic stenosis in high-risk patients regarding mortality, reduction in symptoms and valve hemodynamics [3]. Despite introduction of a wide variety of THVs into the medical sector, with largely favorable results [4, 5] and improved delivery systems, imaging or access site techniques [6–8], one major problem of THVs remains paravalvular leakage (PVL). PVL has a significantly higher incidence following TAVI compared to SAVR and leads to increased mortality if grade of PVL is ≥ moderate (2-year mortality of 30 % in mild aortic regurgitation, 40 % in moderate, 3-year mortality of 60 % in severe aortic regurgitation) [9, 10]. For failing THVs, different strategies had to be developed to control these complications, avoid acute hemodynamic compromise and preserve therapeutic benefits of the TAVI procedure. Besides balloon-post-dilatation, valve-in-valve (ViV) procedures can be taken into consideration [11, 12]. It was shown for the Edwards Sapien XT THV, that it is suitable for ViV procedures in cases of failing CoreValve THV (Medtronic Ltd., Dublin, IRL) with relevant PVL [13]. To date only one case report exists regarding suitability of the Edwards Sapien 3 (S3) THV for ViV treatment of a failing CoreValve THV [14]. In this case series, we report initial experience with the S3 for ViV therapy of different failing THVs in cases of relevant residual PVL.

## Methods

### Preprocedural diagnostics

Routine preprocedural diagnostic work-up included transthoracic (TTE) and transesophageal echocardiography (TEE), coronary angiogram, as well as contrast-enhanced, electrocardiogram-gated multislice computed tomography (CT) scans. Decision for ViV procedure was made after mutual agreement of the local interdisciplinary heart team. All described procedures were performed in a hybrid operating room by an interdisciplinary team consisting of cardiac surgeons and interventional cardiologists.

### Patients

Three procedures were performed using the S3 THV for failing THV between 01/2014 and 12/2014. All patients presented with prohibitive operative risk for SAVR with a mean logistic European System for Cardiac Operative Risk Evaluation I (log EuroSCORE) of 11.89 – 32.63 and log EuroSCORE II of 2.33–19.14. Two of them were male and rang of age was 71–80 years. In one patient scheduled ViV procedure was the third interventional cardiac procedure with a status post CoreValve and Mitraclip implantation. All patients suffered from atrial fibrillation. Detailed clinical data are summarized in Table 1. All procedures which were carried out are in compliance with the Helsinki Declaration.

**Table 1:** Clinical baseline data and initial TAVI procedure information

	Patient no. 1	Patient no. 2	Patient no. 3
Age (y)	80	78	71
Gender	female	male	male
BMI (kg/m^2^)	46.6	21	21.6
Comorbidities	AF, RI, osteoporosis, s/p TKA both sides, AH, NIDDM	AF, 1 VD, prostate cancer, s/p rectum cancer, COPD IV, AH	s/p DES in LAD, 3VD, s/p Mitraclip, AF
logEuroSCORE I (%)	23.92	11.89	32.63
logEuroSCORE II (%)	5.54	2.33	19.14
STS PROM (%)	4.69	3.78	2.744
NYHA	III	III	IV
TTE before index procedure			
LVEF (%)	60	50	30
EOA (cm^2^)	0.9	0.7	0.9
Gradient max/mean (mmHg)	82/41	90/52	30/12
Valve insufficiency	none	none	none
Index procedure			
Implanted THV, size (mm)	Edwards Sapien XT, 26	JenaValve, 27	CoreValve, 29
Symptoms after index procedure	syncope, dyspnea	dyspnea	dyspnea
Time to 2^nd^ procedure (d)	1119	533	814
TTE after index procedure			
EOA (cm^2^)	/	1.9	2.8
Gradient max/mean (mm Hg)	/	27/10	15/9
Valve insufficiency	mild	mild	mild

*Y* years; *BMI* body mass index; *logEuroSCORE* logistic European System for Cardiac Operative Risk Evaluation; *STS PROM* Society of Thoracic Surgeons Predicted Risk of Mortality; *NYHA* New York Heart Association; *TTE* transthoracic echocardiography; *LVEF* left ventricular ejection fraction; *EOA* effective orifice area; *TAVI* transcatheter aortic valve implantation; *THV* transcatheter heart valve; *AF* atrial fibrillation; *RI* renal insufficiency; *TKA* total knee arthroplasty; *AH* arterial hypertension; *NIDDM* non insulin dependent diabetes mellitus; *VD* vessel disease; *s/p* status post; *COPD* chronical obstructive pulmonal disease; *PVL* paravalvular leakage; *SD* standard deviation

### Initial TAVI procedure

Initial TAVI procedures were performed via transfemoral (tf) access in two cases (Edwards Sapien XT, CoreValve) and via transapical (ta) access in one case (JenaValve). Implantation was performed due to severe aortic stenosis in all cases. TTE prior to discharge after index procedure presented mild PVL in all cases with sufficient EOA and pressure gradients and patients were discharged with improved NYHA functional class of ≤ II. Index procedural data are summarized in Table 1.

At an interval of 533–1119 days, patients were readmitted with acute exacerbation of dyspnea or status post syncope. In patient 1 serial TTE examination due to recurrent cardiac decompensation presented severe PVL from one year after the index procedure, which was reluctantly observed and re-intervention was refused due to the high-risk profile. In the other two patients first detection of moderate or severe PVL of the THV led to immediate reinterventions. Severe PVL was demonstrated in all patients on TTE at time of admission. Detailed echocardiography data before ViV procedure are shown in Table 2.

**Table 2:** Preprocedural and postprocedural echocardiography data, Intraprocedural information

	Patient no. 1	Patient no. 2	Patient no. 3
TTE/TEE before 2^nd^ procedure			
LVEF (%)	60	50	30
Gradient max/mean (mm Hg)	18/9	24/12	15/12
EOA (cm^2^)	1.7	2.2	2.3
Valve insufficiency	severe	moderate-severe	severe
ViV procedure	Edwards Sapien XT	JenaValve	CoreValve
Edwards Sapien 3 size (mm)	26	29	29
Procedure time (mm)	95	100	180
Fluoroscopy time (min)	10	11	20
Contrast agent (mL)	121	125	174
TTE discharge			
LVEF (%)	60	50	30
EOA (cm^2^)	1.5	2.5	2.5
Gradient max/mean (mm Hg)	36/16	17/9	14/6
Paravalvular leakage	none	trace	trace
ICU stay (d)	2	1	1
Hospital stay (d)	10	8	20
VARC-2 events at 30 days	Major bleeding	None	TIA

*TTE* transthoracic echocardiography; *TEE* transesophageal echocardiography; *LVEF* left ventricular ejection fraction; *ViV* valve-in-valve; *mm* millimeter; *min* minutes; *mL* milliliter; *EOA* effective orifice area; *ICU* intensive care unit; *VARC-2* Valve academic research consortium; *TIA* transitory ischemic attack; *SD* standard deviation

### Valve-in-Valve implantation procedure

Due to echocardiographic findings and reduced clinical status in all three cases, repeat TAVI was indicated following heart team consensus. Sizing was conducted by reference to CT of the index procedures to reduce radiation exposure with effective annulus sizes (based on CT_eff_ = 2 × √(circumferential area/π) of 23 mm for the Sapien XT patient, 29 mm for the JenaValve patient and 24 mm for the CoreValve patient.

Due to adequate peripheral vasculature as assessed by reconstruction of the planning CT, all procedures were performed via percutaneous tf access following standard procedural protocol. After insertion of a vascular closure system (ProStar XL Percutaneous Vascular Surgical System®, Abbott Vascular, Santa Clara, CA, USA) in the right femoral artery and placement of a pacemaker lead in the RV via the right jugular vein, a guide wire was advanced retrogradely across the THV into the LV. Subsequently the S3 THV was deployed into the THV in a phase of rapid ventricular pacing, with prior positioning of the radio-opaque marker at the height of native the aortic annulus. Intraprocedurally, TEE and aortic root angiography as well as invasive pressure measurements were performed to document adequate THV function.

In two cases a cerebral protection system (Claret Medical, Inc., Santa Rosa, CA, USA) was placed via the right brachial artery into the brachiocephalic trunk and left common carotid artery to protect against possible distal embolization. Intraprocedural data are summarized in Table 2.

Events were recorded according to Valve Academic Research Consortium 2 (VARC-2) standard criteria.

## Results

All patients were successfully implanted with a S3 THV for PVL of previous implanted THV without major complications such as stroke, myocardial infarction due to coronary obstruction or significant aortic regurgitation. Immediate intraprocedural invasive mean pressure measurements showed decline of 10 mmHg to 5 mmHg for S3 in Sapien XT, 10 mmHg to 4 mmHg for S3 in Jenavalve and 21 mmHg to 12 mmHg for S3 in CoreValve. In all patients severity of PVL was reduced to none/trace as documented by intraprocedural TEE, angiography and invasive measurements of hemodynamics. Findings were confirmed by TTE at the time of discharge. Echocardiography results are summarized in Table 2.

**Fig. 1:**
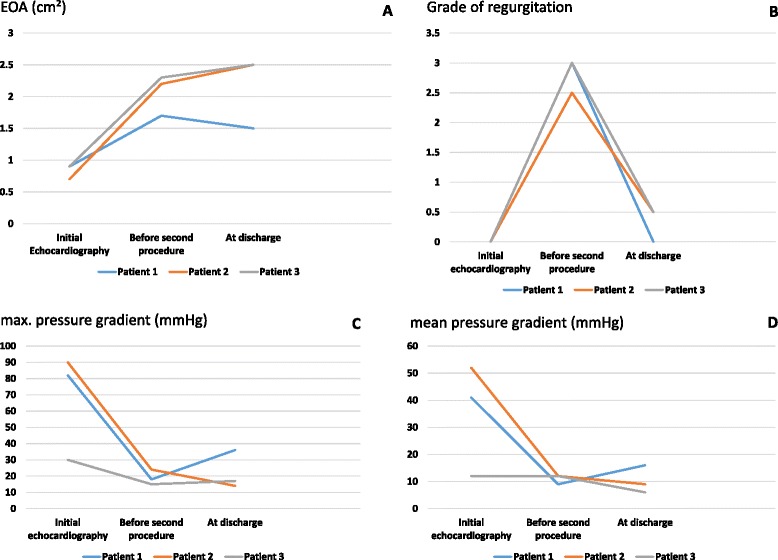
EOA, PVL and max/mean pressure gradients. Graphical overview of development of EOA (**a**), grade of regurgitation (**b**) and max/mean pressure gradients (**c**; **d**) from initial echocardiography before index procedure to discharge after valve-in-valve procedure with the Edwards Sapien 3®. (EOA- effective orifice area; TAVI- transcatheter aortic valve implantation)

Figure 1 shows development of EOA, PVL grade and max/mean pressure gradient over time from initial evaluation until discharge after second procedure. One patient showed slightly decrease of EOA without resulting in clinical symptoms such as dyspnea after the procedure. In all other patients increase in EOA was achieved. Accordingly in TTE prior to discharge mean pressure gradients were lowered in two patients but peak pressure gradients increased in two patients.

**Fig. 2:**
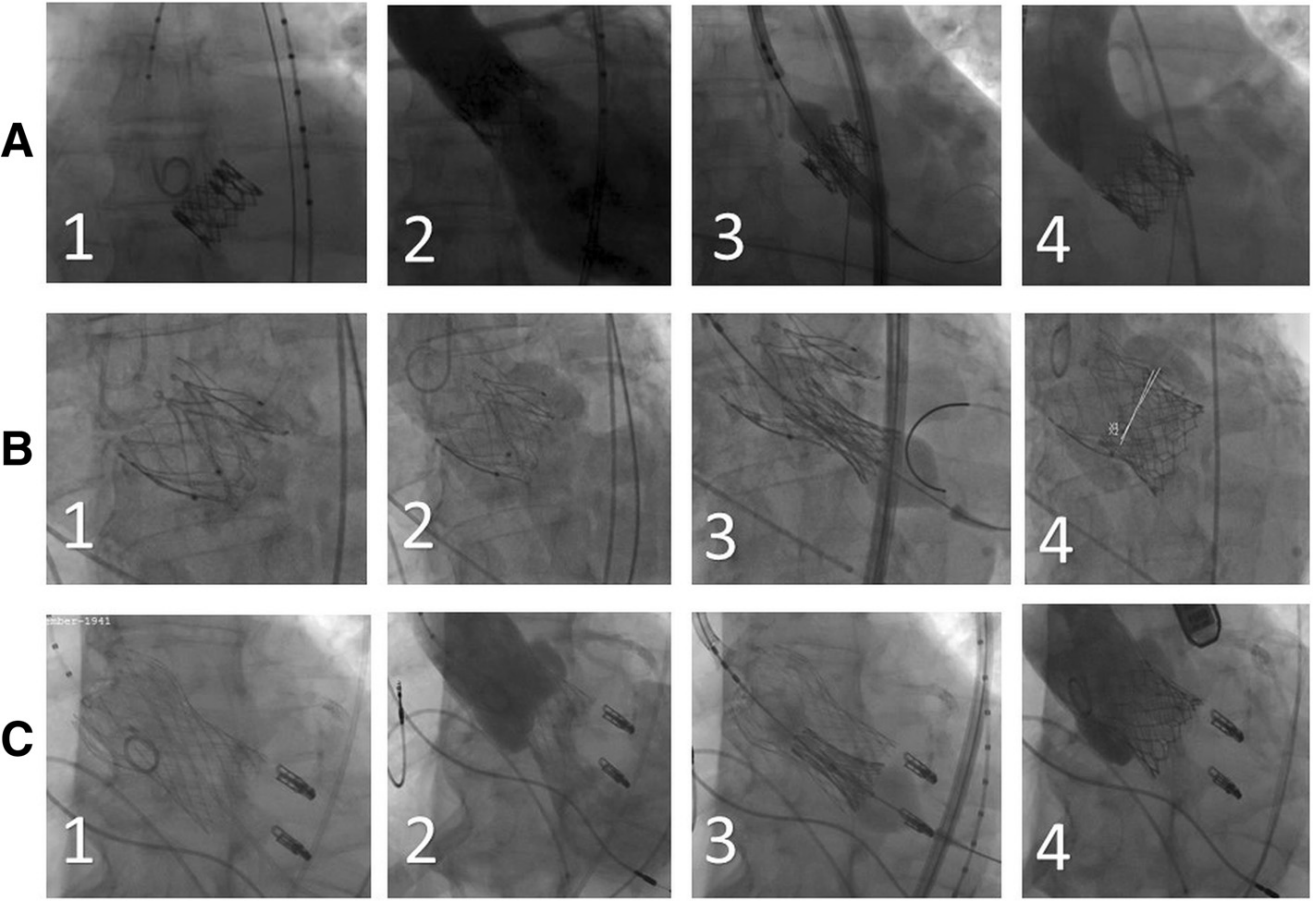
Angiographies of valve-in-valve procedures with the Sapien 3® into Sapien XT®, JenaValve® and CoreValve®. Column (**a**) with angiography of severely regurgitant Sapien XT valve without (1) and with contrast agent (2), deployment of Sapien 3 (3) and final angiography without paravalvular or transvalvular leakage (4) Column (**b**) presents angiography of regurgitant JenaValve without (1) and with contrast agent (2), deployment of Sapien 3 (3) and final angiography with trace paravalvular leakage and no transvalvular leakage (4) Column (**c**) with angiography of regurgitant CoreValve without (1) and with contrast agent (2), deployment of Sapien 3 (3) and final angiography with trace para- and no transvalvular leakage (4)

Intraprocedural angiographies are depicted in Fig. 2.

Three patients presented PVL two to three years after the index procedure and were implanted with an S3 electively with a hospital stay of 8–20 d and an intensive care unit stay of 1–2 d.

According to VARC-2 criteria one major bleeding (access site hematoma requiring transfusion of two units of red blood cells) and one transient ischemic attack (TIA) was observed. TIA became apparent in sensory loss of the right hand of a patient with pre-existing severe stenosis of the left internal carotid artery. Symptoms subsided after 12 h. None of the patients had acute kidney injury or need of renal replacement therapy. All patients were alive at 30 days of follow-up and at the time of 90–530 days after the procedure, respectively.

## Discussion

Reasons for failing THV differ. Eccentric annulus calcification with incomplete stent expansion, aortic or ventricular malpositioning, or malsizing of THV are among the most common causes for PVL [15–17]. Regardless of cause, PVL is responsible for inferior acute and long-term clinical outcomes.

In this work, we presented initial experience with the S3 for ViV therapy of different failing THVs in cases of relevant recurring PVL. ViV procedures were performed safely and reliably for severe PVL in the Sapien XT, the JenaValve and the CoreValve. In self-expandable THV, such as the CoreValve and the JenaValve, radial forces might be too weak in particular cases for full expansion of the stent. When suboptimal expansion occurs ballon-post-dilatation is usually performed. In our cases balloon-post-dilatation was performed at the time of the index procedure but severe PVL still recurred. PVL was increasing over a period of two to three years in these THV to an unacceptable level. As illustrated in Fig. 2, ViV procedures using the S3 led to further expansion of the stent of the initially implanted self-expandable THV and therefore to reduction of PVL to none/trace. Implantation of the S3 at the level of the aortic annulus was suitable and led to satisfying hemodynamic outcome.

The hemodynamic performance of the S3 in Sapien XT showed inferiority resulting in EOA of 1.5 cm^2^ and max/mean pressure gradient of 36/16 mmHg without leading to clinical inferiority. This might be due to implantation of both valves at the height of the aortic annulus and/or the stout stent profile. It might be beneficial, in terms of hemodynamics, to implant THV with supraannular anchoring in those patients to avoid elevated pressure gradients and reduced EOA.

Reasons for failing of initially implanted THV, in terms of PVL, remained unclear. All patients presented only mild PVL in TTE prior to discharge after index procedure without elevated pressure gradients or inadequate EOA. Moreover all initial implanted THV covered the native aortic annulus area and diameter measured prior to the first procedure. Nevertheless, THV used for ViV procedures covered larger aortic annulus diameter and areas.

For detailed diameter and area values see Table 3.

**Table 3:** Overview of native aortic annulus, initial and second THV diameter and area

	Patient no. 1	Patient no. 2	Patient no. 3
Initial annular diameter^a^ (mm)	25.3	26.2	25.6
Initial annular area (mm^2^)	490.3	620.1	530.7
Initial THV, size (mm)	Edwards Sapien XT, 26	JenaValve, 27	CoreValve, 29
Covered annulus area of initial THV^b^ (mm^2^)	415–530	/	415.5–572.6
Covered annulus diameter of initial THV^b^ (mm)	23–26	25–27	23–27
Sapien 3 size (mm)	26	29	29
Covered annulus area of implanted Sapien 3^b^ (mm^2^)	430–546	540–683	540–683
Covered annulus diameter of implanted Sapien 3^b^ (mm)	23.4–26.4	26.2–29.5	26.2–29.5

^a^ based on CT_eff_ = 2 × √(circumferential area/π)

^b^ according to information provided by manufacturer

Thus, reasons for failing are only speculative. Possibly, intra- and postprocedural underestimation of PVL in TTE and TEE examinations, stent-recoiling or measuring errors in MSCT prior to the first TAVI might have been responsible for development of severe regurgitation.

## Conclusions

In conclusion, the S3 was well suitable for ViV therapy in failing THV. Adequate hemodynamic results were obtained. For further assertions concerning functional outcomes long-term results have to be awaited.

## Abbreviations

CT-: Computed tomography
EuroSCORE: European System for Cardiac Operative Risk Evaluation
NYHA: New York Heart Association
PCI: Percutaneous coronary intervention
PROM: Predicted risk of mortality
PVL: Paravalvular leakage
SAVR: surgical aortic valve replacement
STS: Society of Thoracic Surgeons
TA: transapical
TAVI: transcatheter aortic valve implantation
TEE: Transesophageal echocardiography
TF: Transfemoral
THV: Transcatheter heart valve
TIA: transient ischemic attack
TTE: Transthoracic echocardiography
TVL: Transvalvular leakage
VARC: Valve academic research consortium
ViV: Valve-in-valve
